# “It Is My Choice to Control Myself!”: Testing the Mediating Roles of Expectancy and Value in the Association Between Perceived Choice and Self-Control Success

**DOI:** 10.3389/fpsyg.2022.851964

**Published:** 2022-04-05

**Authors:** Tak Sang Chow, Chin Ming Hui, Tiffany Sok U. Siu

**Affiliations:** ^1^Department of Counselling and Psychology, Hong Kong Shue Yan University, Hong Kong, Hong Kong SAR, China; ^2^Wan Chow Yuk Fan Centre for Interdisciplinary Evidence-Based Practice and Research, Hong Kong Shue Yan University, Hong Kong, Hong Kong SAR, China; ^3^Department of Psychology, The Chinese University of Hong Kong, Hong Kong, Hong Kong SAR, China

**Keywords:** self-control, self-determination, value, expectancy, self-efficacy

## Abstract

Past research suggested that when individuals feel that it is their free choice to perform a task, they are more likely to succeed. However, little has been known about the effect of perceived choice of self-control and the psychological processes underlying the benefits of this perception in everyday contexts. To fill this gap, a 7-day experience sampling study (115 college students and 1,725 reported episodes of self-control) was conducted to test whether confidence in sustaining the current self-control activity (*expectancy*) and perceived value of current self-control (*value*) could mediate the link between perceived choice and success in the current self-control activity. The results of multilevel analysis suggested that the perceived choice can boost self-control success by increasing expectancy and value of self-control. These findings add mechanistic understanding of the effect of perceived choice on self-control success.

## Introduction

Self-control, or resisting the appeal of temptations and short-term goals to fulfill the long-term goals, plays a pivotal role in goal pursuits and well-being (Daly et al., 2015; Bernecker and Becker, 2020). It is thus important to study the psychological experiences that sustain the exertion of self-control in everyday contexts. The experience of exerting self-control (e.g., fatigue) can be aversive and undermine task engagement, to the extent that individuals *have to* give up the pursuit of pleasurable temptations and short-term goals. However, this experience can become less aversive when individuals perceive a choice and think that they actually *choose to* do it. As a result, we argue that the perceived choice may increase task engagement and success. Indeed, past research suggests that when individuals feel that it is their free choice to perform a task, they are more likely to engage and finally succeed (Ryan and Deci, 2000). We also tested the mediating roles of expectancy and value. Specifically, we proposed that perceived choice increases self-control success by boosting one's confidence in sustaining self-control (*efficacy expectancy*) and perceived value of self-control (*subjective task value*).

### Perceived Choice of Self-Control

According to the self-determination theory, there are different forms of motivation, ranging from a complete absence of motivation (i.e., *amotivation*) to engagement driven by external rewards (i.e., *extrinsic motivation*) to full engagement driven by inherent enjoyment and interest (i.e., *intrinsic motivation*; Deci and Ryan, 2000). When people experience more autonomy in a task, they are more intrinsically motivated. Therefor*e, autonomous motivation* is desirable. For instance, Williams et al. (1996) found that when individuals experienced autonomous motivation during a weight loss program, they would attend the program more regularly and were more successful in weight loss, even at a 23-month follow-up. Similarly, in academic pursuit, autonomous motivation was associated with less procrastination on doing homework among fifth-grade students (Katz et al., 2014).

Moreover, the perceived availability of choice can facilitate autonomous motivation (Ryan and Deci, 2006). When individuals perceived that it is their own choice to perform a task, they are more likely to have better task engagement and performance. Consistently, Muraven (2008) and Muraven et al. (2008) found that environment that supports the perception of choice can sustain self-control across consecutive tasks. While past studies have examined the influence of perceived choice on task performance in specific domains that require self-control (e.g., academic goal, career goal), none directly explored the impact of perceived choice on everyday self-control success. The present research aims to fill this gap by investigating how perceived choice of self-control influences the resolution of a wider range of everyday-life, naturalistic self-control events. To this end, we adopted an experience sampling approach (Hektner et al., 2007; Bolger and Laurenceau, 2013) which usually ensures better ecological validity and less memory bias (Hofmann et al., 2014; Baumeister et al., 2020). More importantly, the random sampling of daily-life self-control events allows us to have a closer look at how within-person, momentary fluctuation of perceived choice affects the experiences of sustained self-control. In addition, we explored the mediating processes *via* which perceived choice may enhance self-control success.

### The Effect of Perceived Choice of Self-Control: An Expectancy-Value Account

Some earlier studies have attempted to examine how perceived choice may promote self-control. For instance, Legault and Inzlicht (2013) found that the perception of choice may promote self-control by enhancing the neuro-affective responses to self-regulatory failures. Nevertheless, the psychological processes through which perceived choice preserves the endurance of self-control is still far from clear. The present study sought to extend this line of inquiry by testing a motivational process model to explain the ways through which perceived choice facilitates successful resolution of daily-life self-control conflicts.

According to expectancy-value theories, the confidence in attaining a desired goal (*expectancy*) and the importance attached to achieving the goal (*value*) are positioned as the most important motivators of behaviors (Feather, 1990; Wigfield and Eccles, 2000; Beckmann and Heckhausen, 2018). According to Bandura (1997), individuals' confidence in their competence is informed by emotional and physiological states such as perceived task aversiveness, fatigue, and stress. When individuals are confident in their ability to enact appropriate actions to obtain desired outcomes, they are more likely to persist, enjoy, and succeed in a task (Bandura, 1982). Past research found that perceived choice reduces the perceived task aversiveness and stress of performing effortful tasks (Blunt and Pychyl, 2000). When people find the task less aversive, they may have stronger confidence to manage it. Furthermore, perceived choice reflects past enactive mastery experience, which is an important source of efficacy belief (Bandura, 1997). Thus, this perception of choice may increase the confidence in sustaining self-control.

Perceived choice may also enhance subjective task value. Eccles (2009) suggested that individuals attach personal/identity-based importance to the attainment of certain tasks. This attainment value is generated from the perceived fit between characteristics of the tasks with the core identities and self-schema of the individuals. In other words, the value of an activity is high when it affords the manifestation of behaviors or attitudes that are the significant aspects of individuals' central selves. When individuals feel that it is his or her own choice to exert control, they tend to personally endorse and identify with the goal they want to achieve *via* self-control.

There is empirical evidence that perceived autonomy is associated with expectancy and value in goal pursuit. For instance, autonomy support was found to enhance expectancy of successful performance, and perceived importance of course content in both traditional face-to-face teaching (González and Paoloni, 2015) and online learning (Vanslambrouck et al., 2016). Similarly, research on job motivation found that autonomy support facilitates expectancy of career success and perceived value of good performance in workplaces (Wang and Netemeyer, 2002; Saragih, 2015). Given that academic and career pursuits often involve effortful self-control and autonomy support is related to perceived choice, it is likely that expectancy and subjective task value are the underlying motivational processes triggered by perceived choice.

## Method

### Participants

One hundred and twenty-five college students were recruited to participate in the present study. Participants were eligible for the study if they were 18 years old or above and had a smartphone equipped with a touchscreen and a data plan. Eventually, 10 of them dropped out of the study after the initial orientation session so we eventually retained data from 115 participants (81 female; *M*_age_ = 20.52; *SD*_age_ = 1.60). Participants were recruited to participate a 10-week intervention study on social media use. The study consists of three phases of experience-sampling, one before the intervention, and two after. No specific instructions were given before the intervention. Our analyses in this paper were based on the data collected in the pre-intervention phase. Each of them was supposed to answer 5 signals per day for seven consecutive days. Participants received 10 HKD for responding each experience sampling survey. An additional bonus of 100 HKD was awarded to participants who responded to more than 90% of signals throughout the whole period of study.

### Procedure

After obtaining participants' informed consent, a trained research assistant taught participants to respond to the experience sampling signal in the initial orientation session. During the orientation session, the research assistant also explained to participants that self-control conflicts occur when there is a conflict between a concrete, proximal motive, and an abstract, long-term motive. Several examples were given (e.g., resisting the desire to drink alcohol, persisting on writing up a term paper) to clarify the meaning of self-control.

The experience sampling phase would start 1 week after the initial orientation. During the experience sampling phase, participants received five signals per day *via* smartphone using the SurveySignal platform (Hofmann and Patel, 2015). Starting from 10 a.m., participants would receive signals at a random time every 3 h. The time gap between two adjacent signals was at least 40 min. Participants were required to respond to each signal within 30 min otherwise it would expire. In each survey, participants were first asked, “In the past 30 min, have you exerted self-control?.” They were given three options: (1) “yes, resisting a desire” (when you have a desire, you want to fulfill or enjoy something immediately); (2) “yes, persisting on a task”, and (3) “no”. If option 3 was chosen, they would then be directed to a survey about their surrounding environment. After that, those who indicated having experienced a self-control conflict (either resisting a desire or persisting on a task) would answer additional questions such as the nature of the conflict and the duration of the conflict. Central to our research questions, we measured perceived choice of self-control (e.g., “How much did you feel a sense of choice and freedom about resisting [name of the desire] /persisting [name of the task]?”), expectancy (e.g., “How confident were you that you would succeed in resisting [name of the desire]/persisting [name of the task]?”), and subjective task value (e.g., “Overall, how much did you value what you were ultimately trying to attain by resisting [name of the desire] /persisting [name of the task]?”). We also asked participants to indicate the extent to which they successfully resolved the self-control conflict. Participants answered all these questions on 7-point Likert scales (1 = *not at all* to 7 = *extremely*).

### Data Analysis

The collected data had a two-level structure with self-control episodes nested within individuals. To account for the nested structure, multilevel structural equation modeling (MSEM) was employed. Perceived choice, expected ability to sustain self-control, value of overall performance and self-control success were all measured at level 1. All of the variables were centered around each person's mean (i.e., group mean centering; Enders and Tofighi, 2007).

In the present study, we were interested in the mediation effects of expected ability to sustain self-control and value of overall performance in the association between perceived choice and successful self-control. To test these hypotheses, we followed Preacher et al. (2010)'s recommendations to test the 1-1-1 mediation model with random slopes and intercepts using Mplus version 8 (Muthén and Muthén, 1998-2017).

## Results

### Response Rate and Correlation

The total response rate was 84.1% in the present study. Each participant completed 29.1 out of experience sampling surveys on average. Among a total of 3,354 completed surveys, 1,725 (51.43%) included self-control episodes and 1,629 indicated no self-control conflicts (48.57%). Among the 1,725 self-control episodes, 409 episodes (23.71%) indicated the resistance of a desire and 1,316 episodes (76.29%) indicated the persistence of a task. Table 1 presents the descriptive statistics and bivariate correlations among major variables. Overall, perceived choice, expected confidence in sustaining control, and subjective task value all positively associated with self-control success.

**Table 1:** Mean, variance, ICC, and bivariate correlations of all study variables.

**Variables**	**Mean**	**Variance**	**ICC**	**1**	**2**	**3**	**4**
1. Perceived choice	4.67	2.49	0.25	–	0.23^***^	0.14^**^;	0.17^**^;
2. Efficacy	4.85	1.92	0.22	0.39^***^	–	0.45^***^	0.58^***^
3. Value	4.87	1.98	0.32	0.18^**^;	0.66^***^	–	0.45^***^
4. Success	4.92	2.01	0.27	0.28^***^	0.90^***^	0.65^***^	–

*Efficacy, expected ability to exert self-control; Value, value of overall performance; ICC, intraclass correlation. Numbers above the diagonal represent the within-individual correlation while numbers below the diagonal represent the between-individual correlation*.

** *p < 0.01*,

*** *p < 0.001*.

### Does Perceived Choice Enhance Self-Control Success by Increasing Expected Ability to Sustain Self-Control and Value of Overall Performance?

Table 2 presents the coefficients of all paths at both between-person and within-person levels. The total effect of perceived choice on self-control success was not found at the between-person level but at the within-person level. While the indirect effects of both expectancy and value were non-significant at the between-person level, they were both significant at the within-person level. In particular, within-person fluctuation of perceived choice of self-control increased both expectancy and task value, which in turn increased self-control success. After controlling for the mediators, the direct effects of perceived choice on self-control success became non-significant.

**Table 2:** 1-1-1 multilevel mediation model testing the mediation effect of expectancy and value in the association between perceived choice of self-control and self-control success.

**Parameters**	**Estimate**	***SE***	**95% CI**
**Between level**			
Intercept	4.67^*^	2.01	[0.73, 8.61]
Path a_b1_	−0.46	0.28	[−1.00, 0.08]
Path a_b2_	−0.23	0.30	[−0.78, 0.33]
Path b_b1_	0.08	0.24	[−0.40, 0.55]
Path b_b2_	0.00	0.22	[−0.43, 0.43]
Indirect effect 1	−0.13	0.16	[−0.43, 0.18]
Indirect effect 2	−0.02	0.07	[−0.17, 0.12]
Direct effect	−0.78^*^	0.36	[−1.50,-0.08]
Total effect	0.04	0.14	[−0.23, 0.32]
Residual variance success	3.28^***^	0.81	[1.70, 4.86]
Residual variance expectancy	2.29^***^	0.61	[1.09, 3.49]
Residual variance value	2.43^***^	0.65	[1.25, 3.62]
**Within level**			
Path a_w1_	0.23^***^	0.05	[0.13, 0.32]
Path a_w2_	0.14^**^	0.04	[0.06, 0.22]
Path b_w1_	0.46^***^	0.05	[0.36, 0.55]
Path b_w2_	0.28^***^	0.04	[0.19, 0.36]
Indirect effect 1	0.12^***^	0.03	[0.06, 0.18]
Indirect effect 2	0.06^*^	0.02	[0.01, 0.10]
Direct effect	0.01	0.01	[−0.04, 0.05]
Total effect	0.14^***^	0.03	[0.08, 0.21]
Residual variance success	0.71^***^	0.08	[0.55, 0.88]
Residual variance expectancy	1.24^***^	0.12	[1.00, 1.49]
Residual variance value	1.20^***^	0.13	[0.95, 1.44]

*Model is a random slopes model. Path a_b1/w1_= Autonomy → Expected ability to sustain self-control. Path a_b2/w2_ = Autonomy → Value of overall performance. Path b_b1/bw1_ = Expected ability to sustain self-control → Self-control success. Path b_b2/bw2_ = Value of overall performance → Self-control success. Indirect effect 1: Autonomy → Expected ability to sustain self-control → Self-control success. Indirect effect 2: Autonomy → Value of overall performance → Self-control success*.

* *p < 0.05*,

** *p < 0.01*,

*** *p < 0.001*.

## Discussion

The present findings suggested that when individuals feel that it is their choice to exert self-control, they are more likely to succeed in resolving daily-life self-control conflicts. This is consistent with previous laboratory studies which found that interpersonal environments which support perceived choice facilitate success in laboratory self-control tasks (e.g., Stroop task; Muraven, 2008; Muraven et al., 2008). The present study went beyond laboratory demonstration to test the role of perceived choice in naturalistic, daily-life self-control conflicts using an experience sampling approach. This experience sampling approach allows us to examine a wide range of self-control conflicts that occur in participants' immediate environments.

Previous studies have hinted neuro-affective responses to failures, error-related negativity (ERN), as a mediator between autonomous support and performance outcome. For example, autonomous support was found to enhance ERN during self-control failure (Legault and Inzlicht, 2013) and ERN amplitudes was also found to mediate the effect of efficacy belief on performance (Themanson et al., 2011). Our finding might put in an additional understanding on why ERN might account for the effect of autonomous support on performance. The present study suggests that those who feel that it is their choice to exert self-control have stronger confidence to succeed. And higher expectancy might result in more receptive processing of negative performance feedback (Bandura and Cervone, 1983), which is represented on the neural affective level.

The present study also extends the literature by testing a mechanistic account of self-control. The general processes underlying self-control success are still important and unaddressed issues in the literature. The strength model of self-control suggested that self-control draws on a limited, biological resource but this proposition has been challenged on multiple grounds (Lurquin and Miyake, 2017; Baumeister et al., 2018; Friese et al., 2019). Emerging models (e.g., Inzlicht and Berkman, 2015; Kotabe and Hofmann, 2015; Molden et al., 2016) suggest that a shift in motivation is the key processes underlying self-control. Our findings contribute to the search of the motivational processes of self-control. In particular, we found that perceived choice elevates the expectation that one is capable of performing the task and that the task is valuable. These changes in motivational beliefs facilitate self-control success. In this sense, the current study might also help to shed light on how autonomous support mitigate the negative effects of ego-depletion (Moller et al., 2006; Muraven, 2008; Muraven et al., 2008). Past research has shown that after performing a depleting self-control task, the self-efficacy to exert further control is undermined (Chow et al., 2015). Thus, it is possible that perceived choice increases resistance to ego-depletion by counteracting the negative effects of continuous self-control on efficacy belief.

It is important to elucidate the mechanistic processes underlying self-control as it may inform how self-control intervention can be optimized. In most existing self-control training programs, participants are often requested to repeatedly engage in personally meaningless inhibition tasks (e.g., cognitive inhibition tasks like Stroop) without much explanation of the rationale or flexibility to choose among different tasks (Inzlicht and Berkman, 2015; Friese et al., 2017). This approach may create an interpersonal environment that signals coercion instead of autonomy. In light of the present findings, the setting of most self-control training studies, which was characterized by a sense of coercion, might undermine self-efficacy and perceived value of the task. In the future, it may be a worthy endeavor to study the incremental benefits of perceived choice (e.g., providing more choices of self-control tasks) in the traditional self-control training program.

### Limitations and Future Research Directions

One of the caveats of the present study is that it could not eliminate the possibility that perception of success in self-control leads to a stronger perception of choice. Due to the correlational nature of the data, the current study could not establish a strong causal claim between perceived choice and self-control success. Indeed, research on attribution has found that people tend to attribute positive outcome and satisfactory progress to internal cause (Shepperd et al., 2008). This competitive model could not be completely ruled out unless an experimental design was adopted. Nevertheless, past studies have already shown that experimental manipulation of autonomy support has causal impacts on expectancy, value, and task performance in academic, career, and health domains (Curry et al., 1990; Williams et al., 1996). Indeed, we believe that the relationship between perceived choice and self-control success could be dynamic and bidirectional. While perceived choice increases success, successful self-control reinforces the perception of choice in a self-control task.

Furthermore, our measure focuses on a conscious self-report evaluation of different aspects of self-control, but the computation of cost and benefit of a self-control attempt, and self-control success needs not be conscious and intentional (Molden et al., 2016). Bijleveld et al. (2012) have demonstrated that people could unconsciously integrate potential rewards and effort requirements to decide task choice and engagement. They found that participants differentiated high-value and low-value rewards in effort allocation even if rewards were presented subliminally. Thus, there could be an unconscious mechanism underlying self-control. Also, although the experience sampling method enables more accurate assessment of daily experiences that are less prone to memory bias (Scollon et al., 2003), the inherent limitations associated with self-report measures could not be eliminated. For instance, it might be difficult for individuals to distinguish one's perception that the self-control effort was successful and one's confidence in succeeding in that self-control attempt. Also, the present method could not account for self-control conflicts that were not actively reported by the participants. Future research could go beyond explicit self-report measures by using subliminal priming (e.g., Radel et al., 2009), implicit measurements (e.g., Nosek et al., 2011) and behavioral data.

In addition, the sampling of the present research may set constraints on the type of self-control in focus. For instance, while university students might experience more self-control conflicts related to academic pursuit, they were less likely to experience self-control conflicts related to parenting. College students, compared with full-time workers, might also encounter less self-control conflicts related to work and financial management. To further examine the role of perceived choice, self-efficacy and value in self-control, future research could recruit other samples such as working parents.

Lastly, the current study focuses on the attainment value of a task which is the personal/identity-based significance attached by individuals to different activities (Wigfield and Eccles, 2000). Nevertheless, the subjective value of a task could encompass other elements such as the anticipated enjoyment of the task (i.e., intrinsic value), the usefulness of the task in pursuing other important future plans (i.e., utility value), and the perceived cost (e.g., expected effort, negative emotions) of performing the task (see Wigfield et al., 2017; Wigfield and Eccles, 2020, for discussion on these components). Future research could explore if perceived choice of self-control relates to these components of task value. For instance, it is possible that when individuals feel that it is their choice to exert self-control, they would expect the task as less effortful (i.e., lower cost) and more enjoyable (i.e., higher intrinsic value).

## Conclusion

The role of perceived choice on successful task performance has been demonstrated in previous laboratory studies, the present study extends this line of inquiry by testing an expectancy-value process model using an experience sampling strategy. Overall, we find that perceived choice facilitated self-control success by enhancing the confidence in sustaining self-control and perceived value of the current self-control. The present study revealed a detailed mechanistic understanding of the effects of perceived choice in everyday self-control.

## Data Availability Statement

The raw data supporting the conclusions of this article will be made available by the authors, without undue reservation.

## Ethics Statement

The studies involving human participants were reviewed and approved by Human Research Ethical Committee of the Hong Kong Shue Yan University. The participants provided their written informed consent to participate in this study.

## Author Contributions

TC contributed to study conceptualization, research design, leading statistical analyses and interpretation of results, and writing the original text. CH contributed to study conceptualization, research design, and providing comments to refine the manuscript. TS contributed to reviewing and editing the manuscript. All authors contributed to the article and approved the submitted version.

## Funding

This present study was supported by the Faculty Development Scheme of the Research Grants Council of Hong Kong (UGC/FDS15/H03/16).

## Conflict of Interest

The authors declare that the research was conducted in the absence of any commercial or financial relationships that could be construed as a potential conflict of interest.

## Publisher's Note

All claims expressed in this article are solely those of the authors and do not necessarily represent those of their affiliated organizations, or those of the publisher, the editors and the reviewers. Any product that may be evaluated in this article, or claim that may be made by its manufacturer, is not guaranteed or endorsed by the publisher.
